# Effect of anti-IgE therapy on food allergen specific T cell responses in eosinophil associated gastrointestinal disorders

**DOI:** 10.1186/1476-7961-9-7

**Published:** 2011-04-28

**Authors:** Barbara Foster, Shabnam Foroughi, Yuzhi Yin, Calman Prussin

**Affiliations:** 1 Laboratory of Allergic Diseases, National Institute of Allergy and Infectious Diseases, National Institutes of Health, Bethesda, Maryland, USA

## Abstract

**Background:**

Anti-IgE therapy inhibits mast cell and basophil activation, blocks IgE binding to both FcεRI and CD23 and down regulates FcεRI expression by antigen (Ag) presenting cells (APCs). In addition to its classical role in immediate hypersensitivity, IgE has been shown *in vitro *to facilitate Ag presentation of allergens, whereby APC bound IgE preferentially takes up allergens for subsequent processing and presentation. The purpose of this study was to determine whether anti-IgE therapy, by blocking facilitated Ag presentation *in vivo*, attenuates allergen specific Th2 cell responses.

**Methods:**

To test this hypothesis, food allergen specific T cell responses were examined during a 16-week clinical trial of omalizumab in nine subjects with eosinophilic gastroenteritis and food sensitization. Allergen specific T cell responses were measured using carboxyfluorescein succinimidyl ester dye dilution coupled with intracellular cytokine staining and polychromatic flow cytometry. Four independent indices of allergen specific T cell response (proliferation, Ag dose response, precursor frequency, and the ratio of Th2:Th1 cytokine expression) were determined.

**Results:**

Eight of the 9 subjects had measurable food allergen specific responses, with a median proliferation index of 112-fold. Allergen specific T cell proliferation was limited to CD4 T cells, whereas CD8 T cell did not proliferate. Food allergen specific responses were Th2 skewed relative to tetanus specific responses in the same subjects. In contradistinction to the original hypothesis, anti-IgE treatment did not diminish any of the four measured indices of allergen specific T cell response.

**Conclusions:**

In sum, using multiple indices of T cell function, this study failed to demonstrate that anti-IgE therapy broadly or potently inhibits allergen specific T cell responses. As such, these data do not support a major role for IgE facilitated Ag presentation augmenting allergen specific T cell responses *in vivo*.

**Trial registration:**

ClinicalTrials.gov identifier NCT00084097

## Background

FcεRI, the high affinity IgE receptor, is expressed by mast cells and basophils, and upon cross-linking by allergen activates these cells, leading to immediate hypersensitivity[1]. FcεRI is also expressed by dendritic cells (DCs) and monocytes and in this capacity FcεRI may have additional functions beyond immediate hypersensitivity. FcεRI expression by APCs can facilitate the IgE mediated uptake of allergen, ultimately resulting in enhanced antigen presentation and increased T cell activation in vitro[2]. In a similar manner, CD23, the low affinity IgE receptor expressed by B cell can also preferentially capture IgE bound allergen, resulting in enhanced antigen presentation[3]. Such "IgE facilitated antigen presentation" or "antigen capture" can shift the *in vitro *T cell proliferation dose response to allergens by 100-1000-fold[2,3].

Activation of DC by cross-linking FcεRI has a number of additional consequences. Activation of human plasmacytoid DCs (pDCs) via FcεRI induces TNF and IL-10 expression, as well as downregulates TLR9 expression and CpG oligonucleotide induced IFN-α expression[4]. Conversely, activation of pDCs via TLR9 downregulates FcεRI expression. In a similar manner to TLR9, crosslinking of FcεRI inhibits TLR7 mediated IFN-α expression by human pDCs[5]. Furthermore, in both murine and human myeloid DCs, activation by FcεRI cross-linking upregulates CCL28 expression, which is chemotactic for Th2 cells[6,7]. In sum, these findings suggest that FcεRI expression by DCs may have multiple consequences, including augmentation of allergic responses and conversely downregulation of virally induced innate immune responses.

Omalizumab is a humanized anti-IgE monoclonal antibody indicated for use in allergic asthma. Anti-IgE therapy reduces the concentration of circulating free IgE, blocks IgE binding to both FcεRI and CD23, and down regulates surface FcεRI on mast cells, and basophils[8]. Individually or in concert, these actions inhibit mast cell and basophil activation, resulting in a decrease in both early and late phase allergic responses. In addition to its effects on immediate hypersensitivity, omalizumab also downregulates FcεRI expression by dendritic cells[9,10]. Serum from omalizumab treated patients effectively blocks CD23 mediated facilitated allergen binding to B cells[11]. Because of these multifunctional activities of FcεRI and CD23 beyond immediate hypersensitivity and the ability of omalizumab to block IgE binding to both of these receptors, it has been postulated that anti-IgE therapy may have *in vivo *immunomodulatory activity on T cell responses[8].

To test the hypothesis that anti-IgE therapy affects allergen specific T cell responses, we assessed food allergen specific T cell responses in patients with allergic eosinophil associated gastrointestinal disorders (EGID) during a clinical trial of omalizumab. Using carboxyfluorescein succinimidyl ester dye dilution coupled with intracellular cytokine staining and polychromatic flow cytometry[12], four different indices of allergen specific T cell response were measured. Surprisingly, despite the effective IgE blockade, no evidence for omalizumab inhibition of allergen specific responses was found.

## Methods

Nine subjects with allergic EGID were enrolled in a 16-week open label clinical trial of omalizumab, the results of which were previously published[13]. The diagnosis of allergic EGID was based on typical gastrointestinal symptoms, peak tissue eosinophilia of >25 per high-power field (hpf) in stomach or duodenal biopsy specimens, negative work-up for other causes of gut eosinophilia, and evidence of atopy (either ≥ 2 positive skin or in vitro IgE tests out of a panel of 6 common foods [peanut, soy, egg, milk, wheat, shrimp], or a serum IgE ≥ 100 kIU/L). Subject characteristics are detailed in the original report[13]. Subject 5 in the original study had no allergen specific T cell proliferation and was not studied further, leaving 8 subjects for analysis. The National Institute of Allergy and Infectious Diseases (NIAID) Institutional Review Board approved the clinical protocol; all subjects signed informed consent.

For each subject, 2 allergens were selected for study, with a preference for food allergens yielding the highest CFSE proliferation index. Six of the 8 subjects were studied with peanut and shrimp, one with peanut and dust mite, and one with egg yolk and egg white extracts. Food antigens were saline extracts prepared by the investigators as previously described[14]; mixed dust mite extract was obtained commercially (ALK-Abello, Round Rock, TX). Tetanus toxoid was obtained from the Massachusetts Public Health Biological Laboratories, Jamaica Plain, MA. For EC50 dose response experiments, half-log Ag concentrations from 0.3 to 100 μg/ml were used. Samples were analyzed at baseline and again after 16 weeks of omalizumab.

Allergen specific T cell responses were measured using a polychromatic adaptation of published flow cytometry methods utilizing carboxyfluorescein succinimidyl ester (CFSE) dye dilution[12,15].The lymphocyte fraction was obtained by leukaphereis (NIH Clinical Center Department of Transfusion Medicine) and mononuclear cells were isolated using 1.077 ficoll-diatrizoate density gradient separation (Lymphocyte Separation Media-1077 (MO Biomedicals, LLC, Aurora, Ohio), washed twice in HBSS (Invitrogen, Carlsbad, CA) and cryopreserved in liquid nitrogen. Aliquots were thawed, washed twice in RPMI, resuspended in RPMI and stained with 8 μM/L CFSE at 37°C for 10 minutes. CFSE labeling was stopped by adding 5 times the volume of ice cold PBS/1% bovine serum albumin, incubation on ice for 5 minutes, after which the cells were washed an additional 2 times in RPMI. Cells were then resuspended at 5 × 10^5 ^cells/ml in RPMI with 10% autologous serum and cultured at 2 ml per well in a 24 well plate with the indicated concentration of allergen. After 4-5 days, 1 ml of culture supernatant was replaced with fresh media. After 7 days, ionomycin (1 μM), phorbol myristate acetate (20 ng/mL) and brefeldin A (10 μg/mL) were added and the cells incubated an additional 6 hours, at which point DNAse (EMD Chemicals, Gibbstown, NJ) final concentration 3,500 Dornase U/ml was added for an additional 5 minutes. Cells were removed from each well, stained with LIVE/DEAD^® ^Fixable Violet Dead Cell Stain Kit (Invitrogen) according to the manufacturers instructions, washed once in PBS and fixed with 4% paraformaldehyde[16].

**Figure 1:**
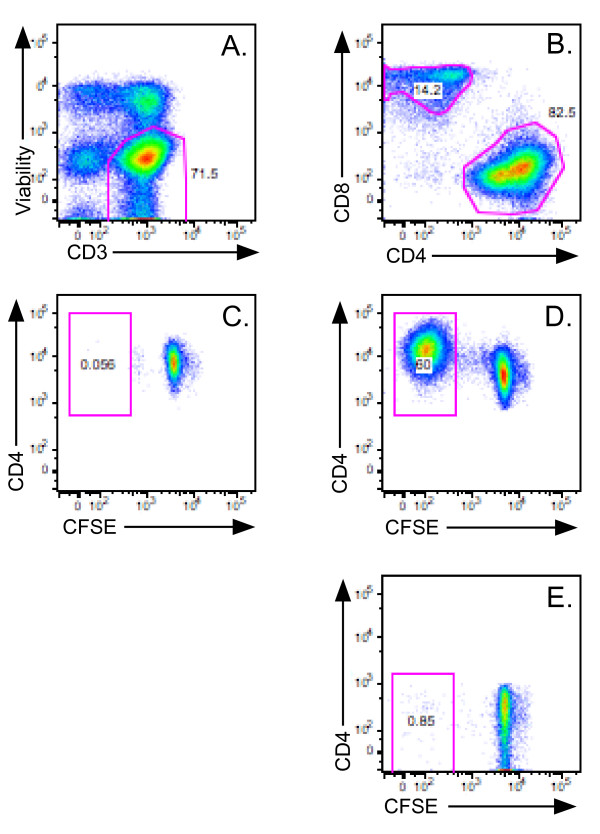
**CFSE determination of allergen specific CD4 T cell responses**. PBMC were activated and stained as per the Material and Methods and then gated on CD3+, violet LIVE/DEAD negative cells (A), and subsequently gated on CD4+, CD8- or CD4-, CD8+ cells (B). After culture with media (C), or peanut antigen extract (D, E), cells were gated on viable (C, D) CD4, or (E) CD8 T cells and CFSE vs. CD4 dotplots were generated.

Fixed cells were then stained for intracellular cytokines using published methods[16]. The following antibody conjugates were used: IL-4 phycoerythrin (PE) [clone 25D2], CD4 PE/Cyanine 5(Cy5) [clone SK3], interferon-γ PE/Cy7 [clone B27], IL-5 allophycocyanin [clone JES1-39D10], tumor necrosis factor (TNF) Alexa 700 [clone Mab 11] (all BD Biosciences); and CD3 allophycocyanin/Cy7 [clone UCHT1] and CD8 PE/Texas Red [clone 3B5](both Invitrogen). Cell doublets were excluded using forward scatter area versus height parameters. Viable CD4 T cells were identified by serial CD3^+^, violet LIVE/DEAD negative and CD4^+^, CD8^- ^gates (Figure 1A, B). Flow cytometry analysis and precursor frequency calculations were performed using FlowJo software (Treestar, Ashland, OR).

**Figure 2:**
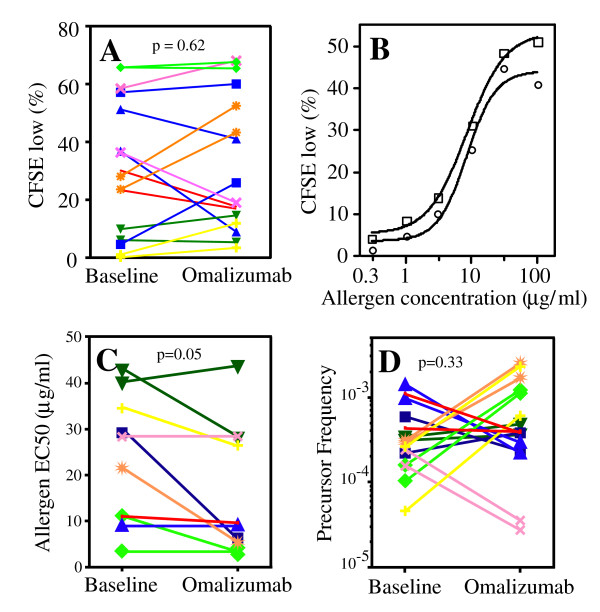
**Effect of anti-IgE therapy on allergen specific T cell proliferation**. (A) Allergen specific CD4 T cell proliferation was measured by calculating the percentage of CFSE^low ^cells and the results compared both at baseline and at completion of a 16-week trial of omalizumab. (B) Example of an allergen dose response to peanut Ag for subject 2, performed at baseline (circles) and at study completion (squares). (C) The EC_50 _for allergen proliferation was compared at baseline and at study completion. (D) The precursor frequency of allergen specific T cells was determined by CFSE dye dilution and compared at baseline and at study completion. Each color/symbol combination represents one subject and one allergen; results in A and D include two allergens examined for each subject.

Proliferation index was calculated as the ratio of CFSE^low ^cells in the Ag vs. media conditions. Pre/post omalizumab calculations of Ag specific CFSE^low ^cells (Figure 2A) were determined using the concentration of Ag yielding maximal proliferation in the "pre" sample. For dose response calculations (Figure 2B, C), the concentration of Ag yielding half maximal proliferation (EC_50_) was determined using Prism software (GraphPad Software, San Diego, CA). Precursor frequency calculations (Figure 2D) were performed using the FlowJo proliferation platform; data from the first and second generation peaks were excluded from these calculations as previously described[17].

Statistical significance was determined using the Wilcoxon signed rank test. Median values were used as a measure of central tendency. In figures, the symbols used to identify individual subjects match those from the original published clinical trial[13].

## Results

To examine the effect of *in vivo *IgE blockade on T cell responses, we first examined T cell proliferation using CFSE dye dilution by determining the percentage of CFSE^low ^cells. Minimal spontaneous proliferation (median = 0.24% CFSE^low ^cells for all donors) was noted in the media control (Figure 1C). In contrast, allergen activated CD4 cells demonstrated substantial proliferation (Figure 1D), with a median of 26.9% CFSE^low ^divided cells. Across all subjects, allergen activation yielded a 112-fold proliferation index over the media control. Minimal allergen driven proliferation was noted in CD8 T cells (Figure 1E), therefore, subsequent analysis was limited to the CD4 subset. Antigen specificity was demonstrated by >90% inhibition of proliferation upon the addition of antibodies against MHC class II (data not shown).

As reported in the original clinical trial, omalizumab effectively blocked IgE as evidenced by an 80% decrease in free IgE, a 75% decrease in basophil FcεRI, a 98.4% decrease in basophil bound IgE, and a >100-fold right shift in the basophil activation dose response[13].

As detailed in the Introduction, IgE may augment allergen specific Th2 responses through a variety of mechanisms. We thus hypothesized that blocking IgE *in vivo *would inhibit Ag presentation of allergens resulting in a decrease in allergen specific T cell activation. As an initial approach to determine the effect of *in vivo *IgE blockade on allergen specific T cell proliferation, we examined the percentage of allergen expanded CFSE^low ^cells at the pre-omalizumab baseline and at the 16-week omalizumab time point. Contrary to our hypothesis, no significant difference was found between the baseline and the 16-week omalizumab time points (29.1% vs. 22.4%, p = 0.62; Figure 2A).

Because *in vitro *IgE mediated antigen facilitated presentation can shift the allergen specific proliferation dose response curve to the left, towards lower antigen doses, we hypothesized that blocking IgE *in vivo *would shift the dose response to the right. To examine this question, we next determined whether omalizumab treatment *in vivo *could shift the allergen proliferation EC_50_. Analyzable sigmoid curves were obtained for all subjects (Figure 2B), with 2 subjects yielding data for two allergens and 6 subjects having analyzable curves for one allergen. Contrary to our hypothesis, anti-IgE therapy was associated with a small 1.5 fold left shift in the EC_50 _towards lower Ag concentration (p = 0.05, Figure 2C).

The frequency of Ag specific T cells is a major determinant of the magnitude of the proliferative response. We thus hypothesized that blocking IgE *in vivo *would decrease the frequency of Ag specific T cells. To address this, we determined whether omalizumab treatment changed the precursor frequency of allergen specific T cells. Contrary to our hypothesis, there was no significant difference in the precursor frequency of allergen specific T cells between the baseline (4.0 × 10^-4^) and the 16-week omalizumab time points (6.5 × 10^-4^, p = 0.33, Figure 2D). Similarly, no significant change was noted in parallel experiments performed with tetanus toxoid (data not shown).

**Figure 3:**
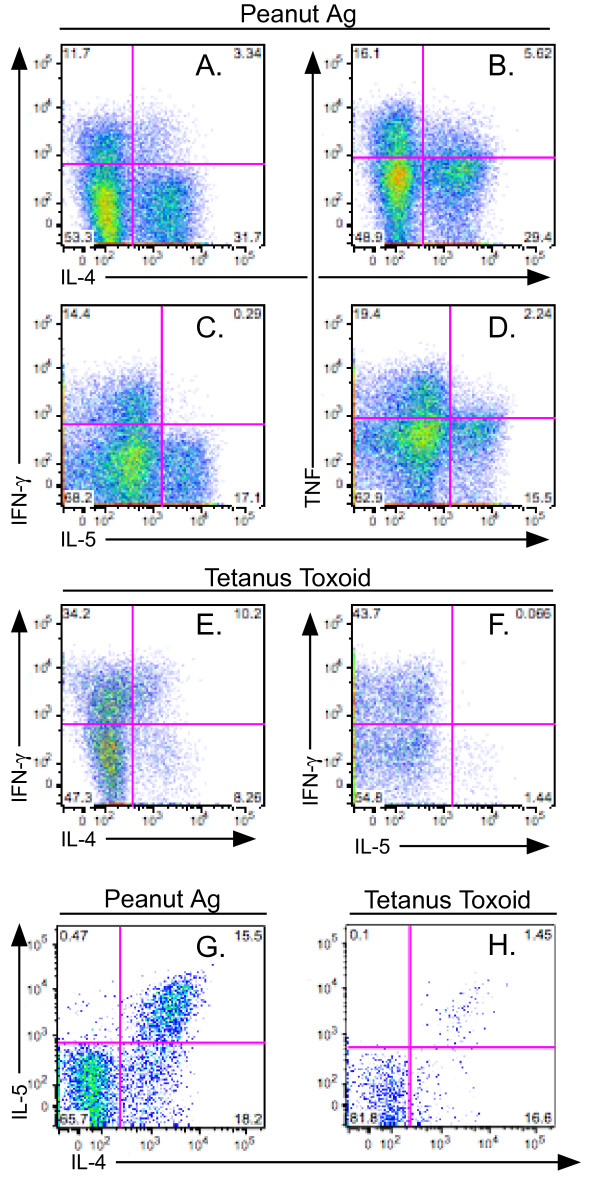
**Allergen specific cytokine staining**. PBMC were activated with peanut Ag (A-D, G) or tetanus toxoid (E, F, H), stained for intracellular cytokines, and after gating on CD4+, CFSE^low ^cells, cytokine dots plots of the noted cytokine pairs were generated.

CFSE dye dilution allows the identification of clonally expanded allergen specific T cells, the cytokine profile of which can be assessed by restimulation in vitro[15]. Food allergen specific T cell responses in EG demonstrated discreet populations of Th1 and Th2 cells (Figure 3). As expected, food allergen specific responses were Th2 biased relative to tetanus toxoid. Notably, the CFSE dye dilution technique identified both IL-5+ and IL-5- subpopulations of allergen specific Th2 cells (Figure 3G, H).

**Figure 4:**
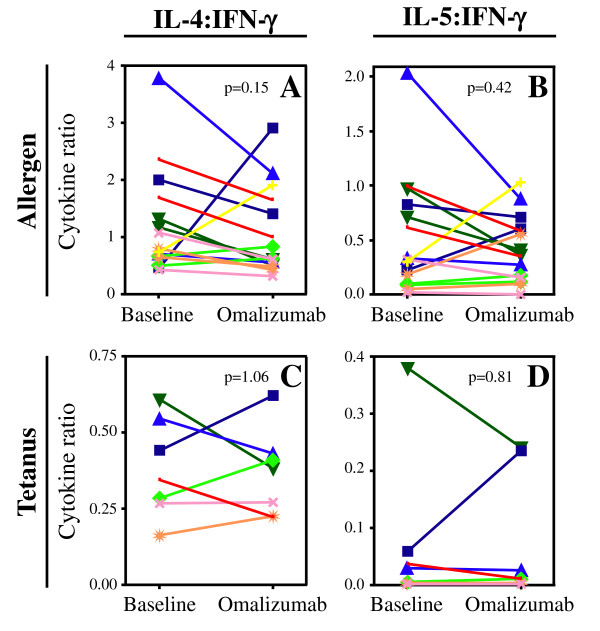
**Effect of anti-IgE therapy on allergen specific T cell cytokines**. The ratio of IL-4:IFN-γ (A, C) and IL-5:IFN-γ (B, D) producing cells were measured in cultures stimulated with either allergen (A, B) or tetanus toxoid (C, D), and compared at baseline and at study completion. Each color/symbol combination represents one subject and one allergen; results in A and B include two allergens examined for each subject.

Through more efficient Ag presentation, mast cell/basophil activation, or antagonism of type 1 IFNs, IgE may augment Th2 allergen specific Th2 skewing. We thus hypothesized that blocking IgE *in vivo *would shift allergen specific T cells responses from a Th2 towards a Th1 bias. To examine this question, we determined the ratio of Th2 to Th1 cytokines in allergen specific CD4 T cells. No significant change was found in the ratios of either IL-4:IFN-γ (baseline 0.81, omalizumab 0.63, p = 0.15), IL-5:IFN-γ (baseline 0.33, omalizumab 0.36, p = 0.42) (Figure 4A, B) or of either Th2 cytokine to TNF-α (data not shown). Similarly, no significant changes were noted in the tetanus toxoid responses (Figure 4C, D).

## Discussion

Inhibition of IgE facilitated Ag presentation by APCs has been hypothesized to be a mechanism by which anti-IgE therapy may decrease allergen specific T cell responses and thus have immunomodulatory activity beyond immediate hypersensitivity[8]. Additionally, IgE may augment Th2 responses via FcεRI mediated activation of mast cells, basophils, and dendritic cells. To address this hypothesis we examined allergen specific T cell responses during a previously reported 16-week clinical trial of omalizumab[13]. Contrary to our original hypothesis, this study failed to demonstrate that anti-IgE therapy had an immunomodulatory or inhibitory effect on food allergen specific T cells responses in EGIDs.

We used an established Ag specific CFSE based proliferative assay[15] to examine four indices of allergen specific T cell response, including proliferation, antigen dose response, precursor frequency, and Th1/Th2 cytokine production. A limitation of this system is that it does not clearly differentiate between changes induced by IgE blocking *in vivo *vs. those occurring in the *in vitro *culture system.

In contrast to our findings, Schroeder and colleagues recently demonstrated that Omalizumab treatment significantly decreased cat allergen specific T cell proliferation by 20-33% and Th2 cytokine expression by 50%[10]. The reasons for the divergent results between the two studies is not clear. Both studies achieved similar levels of *in vivo *IgE blockade and were of similar duration. Notably, the two studies used very different methods to examine allergen specific T cell responses; the previous study used purified CD4 T cell and DC populations, thymidine incorporation and cytokine ELISA, whereas we used unfractionated mononuclear cells and studied proliferation and cytokine expression using flow cytometry. The studies also examined different allergic diseases (cat allergy vs. EGID) and allergen (cat allergen vs. food allergens). Differences in the APC populations, assay systems, T cell to APC ratio, allergen, or disease state are likely factors that account for the divergent results of these two studies.

In contrast to the above, Noga and colleagues examined allergic asthmatic subjects treated with omalizumab for 12 weeks and using ionophore and phorbal ester activated mononuclear cells demonstrated decreased T cell cytokine expression[18]. GM-CSF was the most down regulated cytokine in that study, whereas IL-5 and IFN-γ were not significantly changed. Because that study examined pharmacologically activated rather than allergen specific responses, it is difficult to directly compare those findings to either of the above studies examining allergens.

*In vitro *IgE facilitated Ag presentation shifts the T cell proliferation dose response 100-1000-fold to the left[2,3]. Similarly, in the original report from this trial, omalizumab treatment shifted multiple indices of basophil function between 10 to 150-fold[13]. In contrast to these large magnitude findings, both of the previously published studies above examining omalizumab activity *in vivo *on T cell function showed a relatively modest effect[10,18]. An alternative interpretation of these previous clinical studies is that neither demonstrates an effect size comparable with the *in vitro *data, suggesting that IgE facilitated Ag presentation plays a relatively modest role *in vivo*.

We hypothesized three potential mechanisms whereby anti-IgE therapy could inhibit allergen specific Th2 responses. First, anti-IgE may block IgE facilitated Ag presentation, resulting in decreased allergen specific T cell responses. Through this mechanism, anti-IgE inhibition of Ag presentation could have multiple consequences, including decreased *in vivo *activation and clonal expansion of allergen specific T cells, as well as decreased *in vitro *allergen specific T cell proliferation. Second, anti-IgE may inhibit mast cell and basophil activation *in vivo*,[8], which may result in decreased IL-4 expression, the lack of which could inhibit Th2 cell differentiation. Third, anti-IgE may block FcεRI mediated inhibition of TLR signaling by pDCs, resulting in greater type I interferon expression, which may inhibit Th2 and facilitate Th1 differentiation[19]. A limitation of the current study is that the methods used do not differentiate among these three potential mechanisms.

Recently, in a number of murine model systems, basophils have been shown to be the dominant APC population initiating Th2 responses[20]. However, it is not known whether basophils play a similar role in humans or if omalizumab blocks their APC function.

Greater than 90% of EGID patients respond to an elemental (allergen-free) diet, demonstrating that it is clearly a food allergen driven disease[21,22]. EGID patients do have high rates of atopy and frequently have IgE sensitization to multiple foods[23,24]. However, this food allergen specific IgE typically represents sensitization rather than true IgE mediated food allergy, as most EGID patients do not have anaphylaxis or immediate hypersensitivity clinical reactions to foods. The population used in this study had "allergic" EGID, based on ≥ 2 positive food allergen specific IgE determinations or an elevated total IgE. Notably, the one subject who did not have detectable food specific IgE, did not have measurable food specific T cell responses. Typical for EGID, most of our subjects did not have immediate type hypersensitivity symptoms after eating the foods to which they were sensitized. Because adult EGID differs from conventional anaphylactic food allergy and pediatric EoE, this study's findings may not be generalizable to these latter populations.

This study is notable for several limitations. This study used PBMC, which contains a mixed APC population that may not include specific APC populations that are more IgE dependent. However, if anti-IgE therapy substantially modified T cell responses *in vivo*, such change would be read out by the various endpoints examined, irrespective of the APC population. Notably, this report largely consists of negative results that do not show a statistically significant effect. The substantial results within this work and the academic and ethical issues inherent in non-publication of results supports the value of these findings[25,26]. The statistical and methodological limitations inherent in such small mechanistic studies do no allow us to conclude that omalizumab has absolutely no immunomodulatory effect on allergen specific T cell responses. However, given the multiple T cell endpoints examined in this study, the lack of any data supporting T cell inhibition is striking, particularly when taken in light of the highly significant immunological endpoints from the initial report[13]. This suggests that if omalizumab does modulate T cell responses, the magnitude of such modulation is not of sufficient magnitude to be detected in this study.

We have recently reported that Th2 cells are composed of two major subpopulations obtained using a short term 6-hour assay to identify antigen specific T cells[14]. In this current study, using a different T cell cytokine assay, corresponding IL-5+ and IL-5- allergen specific Th2 subpopulations were found (Figure 3G, H). This Th2 heterogeneity was found in both allergen and tetanus toxoid specific cells, providing further support for it being a generalizable phenomenon.

## Conclusions

In conclusion, examining multiple indices of T cell function, this study failed to demonstrate that anti-IgE therapy has an immunomodulatory or inhibitory effect on allergen specific T cells. As such, these data do not support a major role for IgE facilitated Ag presentation augmenting allergen specific T cell responses *in vivo*.

## Abbreviations

Ag: Antigen; APC: Antigen presenting cell; Cy: Cyanine; CFSE: Carboxyfluorescein succinimidyl ester; EC_50_: Concentration of Ag yielding half maximal proliferation; PBMC: Peripheral blood mononuclear cells; PE: Phycoerythrin;

## Competing interests

The authors declare that they have no competing interests.

## Authors' contributions

BF performed flow cytometric and data analyses, SF performed the clinical trial and participated in the design of the study, YY performed additional data analyses and contributed to writing the manuscript, CP conceived of and designed the clinical trial and study, and wrote the manuscript. All authors read and approved the final manuscript.

## References

[B1] Stone KD Prussin C Metcalfe DD IgE, mast cells, basophils, and eosinophils J Allergy Clin Immunol 2010 125 S73 80 10.1016/j.jaci.2009.11.017 20176269 PMC2847274

[B2] Maurer D Ebner C Reininger B Fiebiger E Kraft D Kinet JP Stingl G The high affinity IgE receptor (Fc epsilon RI) mediates IgE-dependent allergen presentation J Immunol 1995 154 6285 6290 7759866

[B3] van der Heijden FL Joost van Neerven RJ van Katwijk M Bos JD Kapsenberg ML Serum-IgE-facilitated allergen presentation in atopic disease J Immunol 1993 150 3643 3650 8468493

[B4] Schroeder JT Bieneman AP Xiao H Chichester KL Vasagar K Saini S Liu MC TLR9- and FcepsilonRI-mediated responses oppose one another in plasmacytoid dendritic cells by down-regulating receptor expression J Immunol 2005 175 5724 5731 16237063 10.4049/jimmunol.175.9.5724

[B5] Gill MA Bajwa G George TA Dong CC Dougherty II Jiang N Gan VN Gruchalla RS Counterregulation between the FcepsilonRI pathway and antiviral responses in human plasmacytoid dendritic cells J Immunol 2010 184 5999 6006 10.4049/jimmunol.0901194 20410486 PMC4820019

[B6] Grayson MH Cheung D Rohlfing MM Kitchens R Spiegel DE Tucker J Battaile JT Alevy Y Yan L Agapov E Kim EY Holtzman MJ Induction of high-affinity IgE receptor on lung dendritic cells during viral infection leads to mucous cell metaplasia J Exp Med 2007 204 2759 2769 10.1084/jem.20070360 17954569 PMC2118483

[B7] Khan SH Grayson MH Cross-linking IgE augments human conventional dendritic cell production of CC chemokine ligand 28 J Allergy Clin Immunol 2010 125 265 267 10.1016/j.jaci.2009.09.038 19962743 PMC2813948

[B8] Holgate S Casale T Wenzel S Bousquet J Deniz Y Reisner C The anti-inflammatory effects of omalizumab confirm the central role of IgE in allergic inflammation J Allergy Clin Immunol 2005 115 459 465 10.1016/j.jaci.2004.11.053 15753888

[B9] Prussin C Griffith DT Boesel KM Lin H Foster B Casale TB Omalizumab treatment downregulates dendritic cell FcepsilonRI expression J Allergy Clin Immunol 2003 112 1147 1154 10.1016/j.jaci.2003.10.003 14657874

[B10] Schroeder JT Bieneman AP Chichester KL Hamilton RG Xiao H Saini SS Liu MC Decreases in human dendritic cell-dependent T(H)2-like responses after acute in vivo IgE neutralization J Allergy Clin Immunol 2010 125 896 901 e896 10.1016/j.jaci.2009.10.021 20132969

[B11] Klunker S Saggar LR Seyfert-Margolis V Asare AL Casale TB Durham SR Francis JN Combination treatment with omalizumab and rush immunotherapy for ragweed-induced allergic rhinitis: Inhibition of IgE-facilitated allergen binding J Allergy Clin Immunol 2007 120 688 695 10.1016/j.jaci.2007.05.034 17631952

[B12] Roederer M Multiparameter FACS analysis. Curr Protoc Immunol 2002 Chapter 5 Unit 5 8 10.1002/0471142735.im0508s49 18432886

[B13] Foroughi S Foster B Kim N Bernardino LB Scott LM Hamilton RG Metcalfe DD Mannon PJ Prussin C Anti-IgE treatment of eosinophil-associated gastrointestinal disorders J Allergy Clin Immunol 2007 120 594 601 10.1016/j.jaci.2007.06.015 17765756 PMC2768344

[B14] Prussin C Lee J Foster B Eosinophilic gastrointestinal disease and peanut allergy are alternatively associated with IL-5+ and IL-5(-) T(H)2 responses J Allergy Clin Immunol 2009 124 1326 1332 e1326 10.1016/j.jaci.2009.09.048 20004787 PMC2994258

[B15] Turcanu V Maleki SJ Lack G Characterization of lymphocyte responses to peanuts in normal children, peanut-allergic children, and allergic children who acquired tolerance to peanuts J Clin Invest 2003 111 1065 1072 12671056 10.1172/JCI16142 PMC152580

[B16] Foster B Prussin C Coligan JE, Kruisbeek AM, Margulies DH, Shevach EM Unit 6.24, Detection of Intracellular Cytokines by Flow Cytometry In Current Protocols in Immunology 2003 Strober W: Wiley 6.24.21 26.24.16

[B17] Givan AL Fisher JL Waugh MG Bercovici N Wallace PK Use of cell-tracking dyes to determine proliferation precursor frequencies of antigen-specific T cells Methods Mol Biol 2004 263 109 124 14976363 10.1385/1-59259-773-4:109

[B18] Noga O Hanf G Brachmann I Klucken AC Kleine-Tebbe J Rosseau S Kunkel G Suttorp N Seybold J Effect of omalizumab treatment on peripheral eosinophil and T-lymphocyte function in patients with allergic asthma J Allergy Clin Immunol 2006 117 1493 1499 10.1016/j.jaci.2006.02.028 16751018

[B19] Theofilopoulos AN Baccala R Beutler B Kono DH Type I interferons (alpha/beta) in immunity and autoimmunity Annu Rev Immunol 2005 23 307 336 10.1146/annurev.immunol.23.021704.115843 15771573

[B20] Sokol CL Medzhitov R Role of basophils in the initiation of Th2 responses Curr Opin Immunol 2010 22 73 77 10.1016/j.coi.2010.01.012 20144855 PMC3510776

[B21] Spergel JM Eosinophilic esophagitis in adults and children: evidence for a food allergy component in many patients Curr Opin Allergy Clin Immunol 2007 7 274 278 10.1097/ACI.0b013e32813aee4a 17489048

[B22] Chehade M Aceves SS Food allergy and eosinophilic esophagitis Curr Opin Allergy Clin Immunol 2010 10 231 237 10.1097/ACI.0b013e328338cbab 20410819

[B23] Liacouras CA Spergel JM Ruchelli E Verma R Mascarenhas M Semeao E Flick J Kelly J Brown-Whitehorn T Mamula P Markowitz JE Eosinophilic esophagitis: a 10-year experience in 381 children Clin Gastroenterol Hepatol 2005 3 1198 1206 10.1016/S1542-3565(05)00885-2 16361045

[B24] Roy-Ghanta S Larosa DF Katzka DA Atopic characteristics of adult patients with eosinophilic esophagitis Clin Gastroenterol Hepatol 2008 6 531 535 10.1016/j.cgh.2007.12.045 18304887

[B25] Easterbrook PJ Berlin JA Gopalan R Matthews DR Publication bias in clinical research Lancet 1991 337 867 872 10.1016/0140-6736(91)90201-Y 1672966

[B26] Sridharan L Greenland P Editorial policies and publication bias: the importance of negative studies Arch Intern Med 2009 169 1022 1023 10.1001/archinternmed.2009.100 19506169

